# Machine Learning Approach Using Routine Immediate Postoperative Laboratory Values for Predicting Postoperative Mortality

**DOI:** 10.3390/jpm11121271

**Published:** 2021-12-01

**Authors:** Jaehyeong Cho, Jimyung Park, Eugene Jeong, Jihye Shin, Sangjeong Ahn, Min Geun Park, Rae Woong Park, Yongkeun Park

**Affiliations:** 1 Department of Biomedical Sciences, Ajou University Graduate School of Medicine, Suwon 16499, Korea; boyinai03@gmail.com (J.C.); cory66421@ajou.ac.kr (J.P.); 2 Department of Biomedical Informatics, Vanderbilt University School of Medicine, Nashville, TN 37232, USA; eugene.jeong@vanderbilt.edu; 3 Division of Cancer Control & Policy, National Cancer Control Institute, Goyang-si 10408, Korea; sin1125200@naver.com; 4 Department of Pathology, Catholic Kwandong University International St. Mary’s Hospital, Incheon 21431, Korea; vanitasahn@gmail.com; 5 Department of Surgery, Catholic Kwandong University International St. Mary’s Hospital, Incheon 21431, Korea; damian11@ish.ac.kr; 6 Department of Biomedical Informatics, Ajou University School of Medicine, Suwon 16499, Korea

**Keywords:** American Society of Anesthesiologists physical status, surgery, surgical Apgar score

## Abstract

Background: Several prediction models have been proposed for preoperative risk stratification for mortality. However, few studies have investigated postoperative risk factors, which have a significant influence on survival after surgery. This study aimed to develop prediction models using routine immediate postoperative laboratory values for predicting postoperative mortality. Methods: Two tertiary hospital databases were used in this research: one for model development and another for external validation of the resulting models. The following algorithms were utilized for model development: LASSO logistic regression, random forest, deep neural network, and XGBoost. We built the models on the lab values from immediate postoperative blood tests and compared them with the SASA scoring system to demonstrate their efficacy. Results: There were 3817 patients who had immediate postoperative blood test values. All models trained on immediate postoperative lab values outperformed the SASA model. Furthermore, the developed random forest model had the best AUROC of 0.82 and AUPRC of 0.13, and the phosphorus level contributed the most to the random forest model. Conclusions: Machine learning models trained on routine immediate postoperative laboratory values outperformed previously published approaches in predicting 30-day postoperative mortality, indicating that they may be beneficial in identifying patients at increased risk of postoperative death.

## 1. Introduction

The development of new surgical instrumentation and techniques has broadened the applicability of surgical treatment and, consequently, increased the number of patients undergoing surgery. About 310 million surgeries are performed annually worldwide [1]. Numerous studies report that, as access to surgery improves, the incidents of postoperative complications and deaths naturally increase as well [2,3,4]. These events not only have an effect on individual patients’ health outcomes, but also result in greater socioeconomic burden.

Several scoring systems have been devised and validated to predict postoperative mortality by integrating preoperative and intraoperative factors [5]. Dr. Lee Goldman published the revised cardiac risk index called the Lee index, which is a model that assesses the risk of a cardiac event in patients undergoing noncardiac surgery [6,7]. Mascha et al. found that intraoperative hemodynamics is associated with increased 30-day mortality [8]. The Surgical Apgar Score combined with the ASA-PS classification (SASA) scoring system has proved a valuable predictive tool for assessing the surgical risk of complications or death at 30 days using intraoperative hemodynamics and blood loss. These calculators are helpful in determining whether a patient is in optimal medical condition for the planned surgical procedure and in improving postoperative outcomes. However, only a few studies have examined the effect of patients’ conditional changes immediately after surgery on postoperative mortality.

Immediately after major surgical procedures, patients are closely monitored and cared for day and night. Repeated blood tests are used to accurately assess surgical patients’ conditions [9]. To interpret laboratory test results and make a clinical decision, the clinician’s intuition and experience are essential. However, since manually reviewing vast amounts of test results is time consuming and costly, new analysis methods that can reduce the clinician’s burden and identify hidden signs are required. Machine learning (ML) is useful in this situation because it can review a large collection of data and can identify specific trends or patterns that are not apparent to humans [10].

Therefore, the current study aimed to fit and validate a ML model for predicting 30-day mortality using only blood test values measured immediately after surgery. Herein, we expand the process of identifying prognosis with clinical information obtained using three methods immediately after surgery. First, we compared the performance between the SASA scoring system and other ML models, which are 30-day mortality prediction models for patients undergoing surgery in a prospectively collected cohort. Second, the performance of ML models was evaluated using an external validation set. Third, we identified the importance of features used by the model for predicting 30-day mortality.

## 2. Materials and Methods

### 2.1. Study Design and Data

This study includes two cohorts from separate tertiary institutions in South Korea. First, we investigated the VitalDB, which is an open-access de-identified public data set that Seoul National University Hospital collected prospectively from June 2016 to August 2017 [11]. The VitalDB data set is comprised of various intraoperative biosignals along with demographic, operative, and anesthetic data. Moreover, it contains the preoperative and postoperative laboratory values of each subject. Patients who underwent surgery and who have data about postoperative laboratory values, including complete blood count (i.e., white blood cell count, hemoglobin and hematocrit levels, and platelet count), basic metabolic panel (i.e., sodium, potassium, chloride, calcium, phosphate, uric acid, blood urea nitrogen, and creatinine levels), liver function tests (i.e., bilirubin, aspartate aminotransferase, alanine aminotransferase, and alkaline phosphatase levels), serum protein/albumin level, and C-reactive protein levels (CRP), were included.

Routinely collected blood laboratory values immediately after surgery consist of data up to 72 h after surgery. Therefore, patients who died within the first 72 h after surgery were excluded. In addition, patients under the age of 18 or who underwent special surgery such as heart surgery or transplantation were also excluded from this study because they were not only heterogeneous from patients who underwent general surgery, but also received intensive care after surgery. The clinical outcome was 30-day in-hospital mortality excluding 3 days immediately after surgery. The endpoints for assessing 30-day in-hospital mortality for all participants were in-hospital death, 30 days post-surgery, or the last observable day in each database. 

External validation was conducted using data from the Ajou University School of Medicine (AUSOM) database. This database contains information on 2,714,449 patients who visited Ajou University Hospital between February 1994 and May 2020, including their diagnosis, medication prescription, and procedure. Data from the AUSOM database were encoded into the Observational Medical Outcomes Partnership Common Data Model version 5 and de-identification was performed. The cohort used in the external validation comprised patients with major surgical records from the AUSOM database. Major surgery was defined as follows: (1) exposure to endotracheal or intravenous anesthesia and (2) administration of muscle relaxant. Exposure to anesthesia was defined as the use of desflurane, enflurane, isoflurane, sevoflurane, and propofol. The muscle relaxants used were rocuronium, succinylcholine, and vecuronium. Since the training cohort only included patients who underwent general surgery, participants who underwent cardiac surgery, neurosurgery, and transplant surgery at baseline or those who had no immediate postoperative blood test value were excluded. If a patient had multiple test results, the average value was used in the analysis. All details of the validation cohort are presented in Supplementary Material S1. In addition, a patient with at least two missingness in features was dropped. Since most variables of blood test are collected simultaneously, except for the C-reactive protein test, which is not covered by the national health insurance, we considered two missingness were abnormal tests [12].

This study was approved by the Institutional Review Board of Ajou University Hospital (AJIRB-MED-MDB-20-287), and the need for informed consent was waived.

### 2.2. Use of the SASA Scoring System

The SASA score can be calculated using three intraoperative factors: lowest intraoperative heart rate, lowest mean intraoperative blood pressure, and volume of intraoperative blood loss [13,14]. The SASA scoring system combines the Surgical Apgar Score and ASA-PS classification into a single adjusted scale, and the following equation is used [15]:

SASA = Surgical Apgar Score + (6 − ASA physical status classification) × 2

### 2.3. Machine Learning-Based Model Development

We trained the model using the following ML algorithms: deep neural network (DNN), extreme gradient boosting (XGB), least absolute shrinkage and selection operator logistic regression (LASSO), and random forest (RF). For model developments, 75% of data in VitalDB were used for model training and the remaining 25% for testing the training model performances. During the training and testing of the models, 19 blood test values routinely tested immediately after surgery were used as the model predictors. To improve performances, a grid-search pipeline for each model is split into train and validation to identify the best performing hyperparameters with 5-fold cross-validation. The hyperparameter settings of each model were described in Supplementary Material S2.

### 2.4. Statistical Analysis

The characteristics of patients were presented as mean (SD) for continuous variables and number (%) for categorical variables. Between-group differences were compared using the independent two-sample t-test and the χ^2^ test. Two-tailed *p*-values of <0.05 were considered significant. We used the probability score from each ML-based model to calculate the area under the receiver operating characteristic curve (AUROC) and the area under the precision recall curve (AUPRC) for evaluating the predictive performance of SASA scoring system and ML-based models. The AUROC and AUPRC of the external validation cohort were reported. To better understand how nonlinear and tree models work (i.e., XGB and RF models), we evaluated feature contributions to model prediction using SHapley Additive exPlanation (SHAP) value, which is a game-theoretical approach for improving the interpretability of tree-based models [16]. It can explain the global model structure via a combination of local explanations from each ML model prediction. The calculations of SHAP values were performed on all features in the internal test set to evaluate importance and ranking to the final predictive model. The SHAP values were presented as (1) SHAP summary plot, (2) SHAP importance plot, and (3) SHAP dependence plot.

All analyses were performed using R 3.6.2 (R Foundation for Statistical Computing, Vienna, Austria) with the base package and the H2O package (version 3.32.0.1). All source codes for this work are available at https://github.com/abmi/mortalitywithonlylabs (last accessed: 1 December 2021).

## 3. Results

### 3.1. Characteristics of the Cohorts

The VitalDB data set comprised data from 6388 patients who underwent surgery, intraoperative biosignals and other clinical information. The remaining 5940 patients were included in the analysis. The in-hospital mortality rate was 6.8% (n = 402). The median age of the participants was 60 (interquartile range: 50–69) years. The male/female ratio is nearly comparable (50.4% vs. 49.6%). The majority of patients had ASA physical status I (28.4%) or II (61.7%), and the remaining patients had ASA-PS III (9.4%) and IV (0.5%). Most patients underwent general surgical procedures (94.2%), including hepatectomy, pancreatectomy, gastrectomy, colectomy, and thoracic surgical procedures. More than 94% of the procedures were performed under general anesthesia. The median durations of anesthesia use and surgery were 145 (range: 15–1020) and 105 (range: 2–955) min, respectively. Table 1 depicts the patients’ clinical characteristics and intraoperative findings stratified by postoperative mortality. There was a significant difference between the mortality and non-mortality groups. That is, the mortality group was older and had a higher percentage of male participants, lower mean body mass index, and a greater proportion of emergency operations (all *p* < 0.001) than the non-mortality group. Postoperative mortality was significantly associated with the duration of surgery and anesthesia use (all *p* < 0.001) and intraoperative blood loss (*p* = 0.004). The mortality group had a higher ASA-PS score and a lower SASA score than the non-mortality group (both *p* < 0.001).

### 3.2. Profile of Routine Immediate Postoperative Laboratory Values

In total, 3817 patients in VitalDB and 21,640 in AUSOM DB underwent postoperative blood tests within 72 h after non-cardiac surgery, and the results were recorded. Table 2 shows the serum laboratory values, which significantly differed between the non-mortality and mortality groups. In VitalDB, the mortality group had significantly higher blood urea nitrogen, total bilirubin, aspartate transferase, alanine transferase, alkaline phosphatase, and C-reactive protein levels than the non-mortality group. Meanwhile, the non-mortality group had low hemoglobin, hematocrit, sodium, chloride, calcium, albumin, and total protein levels (all *p* < 0.05). In AUSOM DB, the mortality group had a higher white blood cell count and blood urea nitrogen, creatinine, sodium, chloride, uric acid, total bilirubin, aspartate transferase, alanine transferase, alkaline phosphatase, and C-reactive protein levels than the non-mortality group (all *p* < 0.05). Meanwhile, the non-mortality group had low hemoglobin, hematocrit, platelet count, potassium, calcium, albumin, and total protein levels (all *p* < 0.05).

### 3.3. ML Approach for Predicting Postoperative Mortality

First, the performance of each prediction model was evaluated using only data obtained from 2020 patients of VitalDB for whom both intraoperative hemodynamic parameters and immediate postoperative laboratory values were available. Table 3 shows the performances between the SASA scoring system and other ML-based models. The AUROC and AUPRC of the SASA scoring system were 0.73 and 0.06, respectively. The other ML-based models had better performance, with AUROCs and AUPRCs of 0.73–0.82 and 0.24–0.35, respectively. After observing the superiority of ML models over the SASA scoring system, the performance of each ML algorithm was then compared. It was performed on 3817 patients in VitalDB and 21,640 in AUSOM DB with available immediate postoperative laboratory values. Table 4 shows the AUROCs and AUPRCs of the training, test, and external validation performance of the in-hospital mortality models. To evaluate the performance of models in predicting in-hospital mortality, the AUROC (0.75–0.80) and AUPRC (0.26–0.30) were calculated using the test set of the training cohort. Based on the result of the external validation, the AUROC and AUPRC values were 0.70–0.82 and 0.09–0.13, respectively. The RF model had the best performance with an AUROC of 0.82 and AUPRC of 0.13 in the external validation. A calibration plot is presented in Supplementary Material S3.

### 3.4. Importance of Model Feature

**Figure 1:**
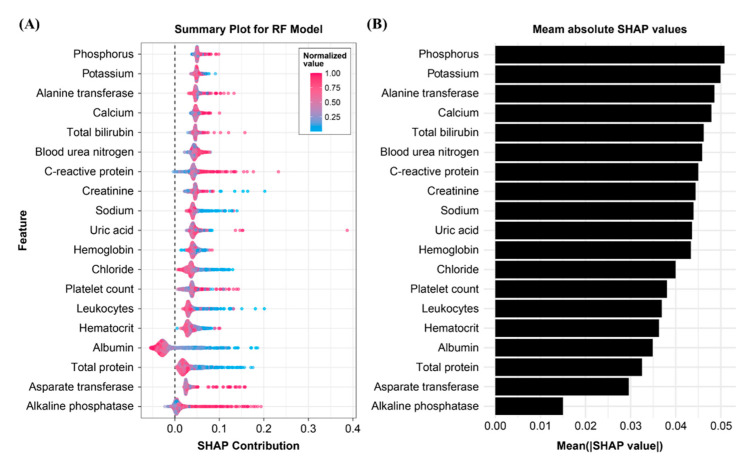
SHAP bee swarm plots and feature importance: (**A**) in the honey bee swarm plot, each point corresponds to a laboratory value observed in an individual person; (**B**) mean absolute SHAP values suggest a rank order for feature importance in the 30-day mortality.

**Figure 2:**
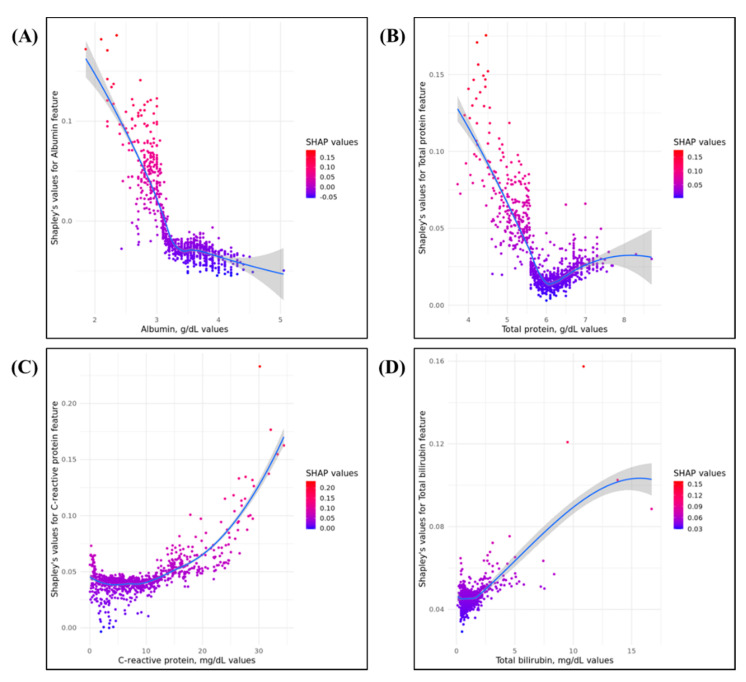
SHAP feature dependence plots for four variables: (**A**,**B**) higher values of albumin and total protein are associated with higher risk of 30-day postoperative mortality; (**C**,**D**) higher C-reactive protein and total bilirubin are associated with lower risk of 30-day postoperative mortality.

The mean absolute SHAP values were calculated for the RF model in the internal validation cohort to evaluate the feature importance. Figure 1 shows the summary plot. Phosphorus level was the most important factor in predicting 30-day in-hospital mortality after surgery, followed by potassium and alanine transferase levels. By contrast, alkaline phosphatase level had the lowest contribution to the model, followed by aspartate transferase, serum total protein, and albumin levels. Most features had positive contribution to the developed RF model, except for albumin and alkaline phosphatase levels. Figure 2 shows the SHAP dependency plots for albumin, bilirubin, CRP, and total protein levels. As shown in Figure 2A,B, low albumin and total protein levels were associated with a higher risk of 30-day postoperative mortality. In contrast, a high CRP level can be associated with a higher risk of mortality (Figure 2C). Most patients had bilirubin levels of <5 mg/dL. Although an increased bilirubin level is associated with high mortality risk, the impact of the feature on the model is difficult to assess.

## 4. Discussion

This retrospective cohort study developed five ML models for predicting 30-day postoperative mortality using only blood test results. The RF model had the best performance in the external validation, with an AUROC of 0.82 and AUPRC of 0.13. The developed RF model outperformed other models (i.e., DNN, XGBoost, and LASSO including SASA score), which are widely known as useful for predicting postoperative mortality. We emphasized several important findings, along with their clinical implications for postoperative patient management. First, we developed a 30-day mortality prediction model that retains training outcomes in both the prospective data set and external validation experiments for patients undergoing surgical intervention.

The advent of modern surgical instrumentation and techniques and the development of anesthesia aim to improve the care of patients undergoing surgery. Further, the continuous progress in critical care has made an important contribution in improving the prognosis of patients after surgery. As a result of these efforts, the postoperative mortality rate has been decreasing significantly for decades [17]. Postoperative death is no longer an inevitable risk that must be endured. Rather, it is a problem that must be prevented [18,19]. Recent studies have proposed the use of various models for predicting postoperative mortality [5,6,7,8,14,15,20], which can help us determine whether to proceed with surgery for each patient. However, regardless of how excellent a predictive model is, it is hard to perfect, and unexpected problems are encountered during the postoperative period. Nevertheless, re-evaluation of a patient’s condition immediately after surgery is more complicated than preoperative assessment. We have applied the ML approach in creating a sophisticated method using routine laboratory values for predicting postoperative mortality in patients undergoing surgery. This novel approach can be used at a patient’s bedside and can be implemented for clinical decision making.

The Surgical Apgar Score (SAS) uses a 10-point scoring system that is based on a patient’s estimated blood loss, the lowest mean arterial pressure, and lowest heart rate during a surgical procedure [13]. Patients with a low SAS had higher rates of postoperative life-threatening complication or death [21,22]. A new surgical scoring system called SASA has been proposed by combining both SAS and ASA-PS. A past study showed a higher predictive ability of the SASA for postoperative mortality than that of the SAS or ASA-PS alone [15]. As with the result of previous studies, the SASA scoring system was demonstrated to be useful for predicting mortality in this study. However, the predictive performance of SASA scoring system was lower than that of the machine learning models using immediate postoperative laboratory values. Deterioration of laboratory values immediately after surgery would better reflect the change in the patient’s perioperative condition.

Remarkably, immediate postoperative serum phosphorus levels were found to be the strongest prognostic indicator for 30-day postoperative mortality in this study. Recent studies have shown an independent association between serum phosphorus level and mortality risk in patients with chronic kidney disease [23,24]. Abnormal serum phosphorus level has been considered an independent risk factor for mortality in patients admitted to intensive care units [25], and a biomarker for predicting acute kidney injury after cardiac surgery in children [26].

In patients undergoing elective surgery, serum albumin levels have been considered a prognostic factor of postoperative morbidity and mortality [27]. A study showed that preoperative albumin levels of <3 g/dL can predict the increased risk of developing serious complications within 30 days after surgery [28]. Another recent prospective study showed that a decrease in serum albumin concentration of ≥10 g/L during the immediate postoperative period was associated with a threefold increased risk of postoperative morbidity [29]. As reported in previous studies, our current study revealed that serum albumin level is the strongest contributor for predicting postoperative mortality. A decline in the serum albumin level after surgery may reflect the extent of postsurgical stress response.

Changes in CRP were also found to be associated with postoperative outcomes. A recent study demonstrated that postoperative CRP levels predict immediate and long-term mortality in patients with operable lung cancer [30]. The results of this study support previous findings.

The current study had a few limitations that must be addressed. This multicenter study reported that the ML model is effective for predicting postoperative mortality. However, it was an observational study with a potential risk of selection bias, which we tried to mitigate by using an independent external validation data set. The lack of documentation about the causes of postoperative deaths is another potential limitation, as some may have been completely unrelated to surgery. The type of surgery plays an important role in the prognosis after surgery. However, in this study, subgroup analysis according to the type of surgery was not performed. Traditionally, surgeons measure surgical success in terms of 30-day mortality and morbidity. Hence, patients who died between the 3rd and 30th postoperative days were included in the postoperative mortality group. Patients who died after the 30th postoperative day due to surgical complications must have been mis-selected in the survival group, which could have led to some analysis errors. Nevertheless, a large patient population was included in this study, and it might have offset the limitations. In addition, we used the mean values of repeated laboratory measurements to train the model, rather than evaluating the trend. Future investigation should consider evaluating and using the trend of the lab results of each patient.

Clinicians request routine laboratory examinations repeatedly, including metabolic panels and complete blood count, to assess the status of their patients who underwent surgical procedures. However, the interpretation of results is fragmentary, and their influence on management is transient. Important clues about changes in the patients’ conditions could be missing. Machine learning models can help to find unrecognized changes in surgical patients’ conditions. To enhance the clinical applicability of these models, further validation is essential and is currently ongoing.

## 5. Conclusions

This study reveals the usefulness of a machine learning model based on blood test values measured immediately after surgery in predicting 30-day in-hospital mortality. We consider this study to be a preliminary study, and a follow-up study is planned to provide personalized risk management to patients undergoing surgery.

## Figures and Tables

**Table 1:** Patient characteristics stratified by postoperative mortality in VitalDB.

	Postoperative Mortality		*p* Value
	No (n = 5538)	Yes (n = 402)	
Mean age, year	58.0 ± 14.1	64.1 ± 13.4	<0.001
Gender, no. (%)			<0.001
Female	2808 (50.7%)	139 (34.6%)	
Male	2730 (49.3%)	263 (65.4%)	
Mean body mass index, kg/m^2^	23.5 ± 3.5	21.8 ± 3.7	<0.001
ASA physical status classification			<0.001
1	1622 (29.3%)	33 (8.2%)	
2	3364 (60.7%)	230 (57.2%)	
3	434 (7.8%)	118 (29.4%)	
4	15 (0.3%)	12 (3.0%)	
N/A	103 (1.9%)	9 (2.2%)	
Type of Surgery, no. (%)			<0.001
Elective	4977 (89.9%)	300 (74.6%)	
Emergency	561 (10.1%)	102 (25.4%)	
Department			
General surgery	4189 (75.6%)	320 (79.6%)	
Gynecology	223 (4.0%)	5 (1.2%)	
Thoracic surgery	1011 (18.3%)	76 (18.9%)	
Urology	115 (2.1%)	1 (0.2%)	
Type of Anesthesia, no. (%)			
General	5220 (94.3%)	379 (94.3%)	
Sedation/Analgesia	52 (0.9%)	19 (4.7%)	
Spinal	266 (4.8%)	4 (1.0%)	
Duration of operation, min.	126.1 ± 93.2	145.5 ± 106.8	<0.001
Duration of anesthesia, min.	166.0 ± 101.4	187.0 ± 114.0	<0.001
Intraoperative monitoring			
Minimal heart rate, beats per min.	46.7 ± 18.7	49.1 ± 24.5	0.142
Minimal mean BP, mmHg	64.7 ± 13.4	63.4 ± 14.6	0.137
Estimated blood loss, mL	279.6 ± 674.9	686.3 ± 2160.7	0.004
SASA score	16.0 ± 2.3	14.1 ± 2.6	<0.001

ASA, the American Society of Anesthesiologists.

**Table 2:** Immediate routine postoperative laboratory values in patients with or without postoperative mortality.

	VitalDB Cohort			AUSOM Cohort		
	Postoperative Mortality		*p* Value	Postoperative Mortality		*p* Value
	No (n = 3523)	Yes (n = 294)		No (n = 20,954)	Yes (n = 686)	
White blood cell count, ×1000/mcL	10.2 ± 3.4	10.2 ± 4.3	0.997	10.4 ± 3.8	11.9 ± 5.6	<0.001
Hemoglobin, g/dL	11.6 ± 1.8	10.5 ± 1.7	<0.001	11.5 ± 1.8	10.4 ± 1.6	<0.001
Hematocrit, %	35.5 ± 5.2	31.8 ± 5.1	<0.001	34.2 ± 5.2	30.8 ± 4.7	<0.001
Platelet count, ×1000/mcL	202.7 ± 74.4	210.3 ± 112.2	0.255	208.4 ± 87.8	155.2 ± 90.4	<0.001
Blood urea nitrogen, mg/dL	13.5 ± 8.4	16.3 ± 10.8	<0.001	12.5 ± 7.2	20.5 ± 14.1	<0.001
Creatinine, mg/dL	0.9 ± 1.0	1.0 ± 0.9	0.434	0.9 ± 0.8	1.3 ± 1.3	<0.001
Sodium, mmol/L	138.1 ± 2.6	137.1 ± 3.5	<0.001	138.9 ± 2.9	141.4 ± 6.4	<0.001
Potassium, mmol/L	4.0 ± 0.4	4.1 ± 0.4	0.602	4.0 ± 0.4	3.8 ± 0.5	<0.001
Chloride, mmol/L	102.7 ± 3.0	102.2 ± 3.9	0.014	103.3 ± 3.7	106.1 ± 6.7	<0.001
Calcium, mg/dL	8.4 ± 0.5	8.2 ± 0.5	<0.001	8.3 ± 0.7	7.8 ± 0.7	<0.001
Phosphorus, mg/dL	2.9 ± 0.8	3.0 ± 0.8	0.289	3.2 ± 0.8	3.2 ± 1.1	0.781
Uric acid, mg/dL	3.4 ± 1.5	3.4 ± 1.6	0.717	3.7 ± 1.5	3.9 ± 2.1	0.004
Total bilirubin, mg/dL	1.1 ± 1.1	1.6 ± 3.0	0.007	0.9 ± 1.1	1.9 ± 3.2	<0.001
Asparate transferase, IU/L	52.8 ± 204.4	129.8 ± 542.6	0.016	53.2 ± 134.0	161.0 ± 460.5	<0.001
Alanine transferase, IU/L	51.5 ± 166.2	92.0 ± 264.8	0.01	43.1 ± 90.9	85.2 ± 219.8	<0.001
Alkaline phosphatase, IU/L	61.0 ± 29.0	82.1 ± 56.9	<0.001	84.0 ± 101.2	93.9 ± 95.9	0.012
Albumin, g/dL	3.4 ± 0.4	3.0 ± 0.4	<0.001	3.5 ± 0.5	3.0 ± 0.5	<0.001
Total protein, g/dL	6.0 ± 0.7	5.5 ± 0.7	<0.001	5.8 ± 0.8	5.1 ± 0.9	<0.001
C-reactive protein, mg/dL	8.3 ± 6.1	11.9 ± 7.6	<0.001	5.9 ± 6.2	12.6 ± 9.7	<0.001

**Table 3:** Performance metrics between SASA scoring system and other machine learning-based models using VitalDB.

	Candidate Models				
	SASA	LASSO	DNN	RF	XGB
AUROC	0.73	0.73	0.84	0.74	0.82
AUPRC	0.06	0.26	0.35	0.24	0.30

AUROC, area under receiver operating curve; AUPRC, area under precision recall curve; SASA, Surgical Apgar Score combined with the ASA-PS classification; LASSO, least absolute shrinkage and selection operator logistic regression; DNN, deep neural network; RF, random forest; XGB, extreme gradient boosting.

**Table 4:** Performance metrics between developed models developed with only postoperative blood test values.

		Machine Learning Models			
		LASSO	DNN	RF	XGB
AUROC	Train	0.81	0.82	0.77	0.90
	Test	0.77	0.79	0.75	0.80
	External validation *	0.70	0.72	0.82	0.75
AUPRC	Train	0.35	0.31	0.31	0.53
	Test	0.26	0.27	0.29	0.30
	External validation *	0.09	0.08	0.13	0.09

AUROC, area under receiver operating curve; AUPRC, area under precision recall curve; SASA, Surgical Apgar Score combined with the ASA-PS classification; DNN, deep neural network; RF, random forest; XGB, extreme gradient boosting. * External validation was performed on AUSOM DB, while train and test were performed on VitalDB.

## Data Availability

All detailed data included in the study are available upon appropriate request by contact with the corresponding author.

## Supplementary Materials

The following are available online at https://www.mdpi.com/article/10.3390/jpm11121271/s1, Supplementary Material S1: Cohort definition and concept sets, Supplementary Material S2: The settings and results of experimental models, Supplementary Material S3: A calibration plot comparing the predicted probability computed by the random forest model with the fraction of observed outcome.
