# Supplementary material for: Machine Learning Approach Using Routine Immediate Postoperative Laboratory Values for Predicting Postoperative Mortality
Source: J Pers Med. 2021 Dec 1;11(12):1271. doi: 10.3390/jpm11121271 (PMC8706001; doi:10.3390/jpm11121271)
Supplement: Supplementary file 1 [file jpm-11-01271-s001.zip › jpm-1468706-SI.pdf]

Supplementary Material S1: Cohort definition and concept sets.

[JC] Surgery cohort

Initial Event Cohort

People having any of the following:

- a drug exposure of flurane[3]
- a drug exposure of propofol[5]

with continuous observation of at least 0 days prior and 0 days after event index date, and limit initial events to: **all events per person.**

Inclusion Rules

Inclusion Criteria #1: Muscle relaxant use
Having all of the following criteria:

- at least 1 occurrences of a drug exposure of Muscle relaxant [4]

  where event starts between 1 days Before and 1 days After index start date
  occurring within the same visit

Inclusion Criteria #2: cardiac or brain surgery
Having all of the following criteria:

- at most 0 occurrences of a procedure of cardiac or brain surgery[2]

  where event starts between 7 days Before and 7 days After index start date

Inclusion Criteria #3: transplant
Having all of the following criteria:

- at most 0 occurrences of a procedure of transplant[7]

  where event starts between 7 days Before and 7 days After index start date

Limit qualifying cohort to: **all events per person.**

End Date Strategy

Date Offset Exit Criteria

This cohort defintion end date will be the index event's start date plus 0 days

Cohort Collapse Strategy:

Collapse cohort by era with a gap size of 0 days.

Appendix 1: Concept Set Definitions

1. cadaveric

Show 10 entries

Search:

Concept Id	Concept Name	Domain	Vocabulary	Excluded	Descendants	Mapped
No data available in table						

Showing 0 to 0 of 0 entries

PreviousNext

2. cardiac or brain surgery

Show entries

Search:

Concept Id	Concept Name	Domain	Vocabulary	Excluded	Descendants	Mapped
2110581	Cranioplasty for skull defect with reparative brain surgery	Procedure	CPT4	NO	YES	NO
4010257	Valvuloplasty of aortic valve	Procedure	SNOMED	NO	YES	NO
4042676	Procedure on pericardium	Procedure	SNOMED	NO	YES	NO
4081578	Coronary artery atherectomy	Procedure	SNOMED	NO	YES	NO
4095407	Replacement of aortic valve	Procedure	SNOMED	NO	YES	NO
4098191	Replacement of tricuspid valve	Procedure	SNOMED	NO	YES	NO
4106548	Aortocoronary bypass of one coronary artery	Procedure	SNOMED	NO	YES	NO
4149930	Pericardiocentesis	Procedure	SNOMED	NO	YES	NO
4150819	Operative procedure on coronary artery	Procedure	SNOMED	NO	YES	NO

Concept Id	Concept Name	Domain	Vocabulary	Excluded	Descendants	Mapped
4163951	Electrocardiographic procedure	Procedure	SNOMED	YES	YES	NO

Showing 1 to 10 of 27 entries

Previous123Next

3. flurane

Show

Search:

Concept Id	Concept Name	Domain	Vocabulary	Excluded	Descendants	Mapped
743196	enflurane	Drug	RxNorm	NO	YES	NO
782043	isoflurane	Drug	RxNorm	NO	YES	NO
782046	isoflurane 999 MG/ML	Drug	RxNorm	NO	YES	NO
782047	isoflurane 999 MG/ML Inhalation Solution	Drug	RxNorm	NO	YES	NO
19002770	desflurane	Drug	RxNorm	NO	YES	NO
19002793	desflurane 1 ML/ML	Drug	RxNorm	NO	YES	NO
19002796	desflurane 1000 MG/ML Inhalation Solution	Drug	RxNorm	NO	YES	NO
19039298	sevoflurane	Drug	RxNorm	NO	YES	NO
40042723	Enflurane Inhalant Solution	Drug	RxNorm	NO	YES	NO
40050254	Isoflurane Inhalant Solution	Drug	RxNorm	NO	YES	NO

Showing 1 to 10 of 14 entries

Previous12Next

4. Muscle relaxant

Show entries

Search:

Concept Id	Concept Name	Domain	Vocabulary	Excluded	Descendants	Mapped
836208	succinylcholine	Drug	RxNorm	NO	YES	NO
19003953	rocuronium	Drug	RxNorm	NO	YES	NO
19012598	vecuronium	Drug	RxNorm	NO	YES	NO

Showing 1 to 3 of 3 entries

Previous1Next

5. propofol

Show entries

Search:

Concept Id	Concept Name	Domain	Vocabulary	Excluded	Descendants	Mapped
753626	propofol	Drug	RxNorm	NO	YES	NO

Showing 1 to 1 of 1 entries

Previous1Next

6. Surgery

Show entries

Search:

Concept Id	Concept Name	Domain	Vocabulary	Excluded	Descendants	Mapped
45888085	Surgery	Procedure	CPT4	NO	YES	NO

Showing 1 to 1 of 1 entries

Previous1Next

7. transplant

Show entries

Search:

Concept Id	Concept Name	Domain	Vocabulary	Excluded	Descendants	Mapped
2109321	Liver allotransplantation, orthotopic, partial or whole, from cadaver or living donor, any age	Procedure	CPT4	NO	YES	NO
4208341	Solid organ transplant	Procedure	SNOMED	NO	YES	NO
4322471	Transplant of kidney	Procedure	SNOMED	NO	YES	NO

Showing 1 to 3 of 3 entries

Previous1Next

Supplementary Material S2: The settings and results of experimental models.
RF

$keep_cross_validation_models
[1] TRUE

$keep_cross_validation_predictions
[1] FALSE

$keep_cross_validation_fold_assignment
[1] FALSE

$score_each_iteration
[1] FALSE

$score_tree_interval
[1] 0

$fold_assignment
[1] "Random"

$ignore_const_cols
[1] TRUE

$balance_classes
[1] FALSE

$max_after_balance_size
[1] 5

$max_confusion_matrix_size
[1] 20

$ntrees
[1] 100

$max_depth
[1] 20

$min_rows
[1] 1

$nbins

```
[1] 30

$nbins_top_level
[1] 1024

$nbins_cats
[1] 1024

$r2_stopping
[1] 1.797693e+308

$stopping_rounds
[1] 0

$stopping_metric
[1] "AUC"

$stopping_tolerance
[1] 0.001

$max_runtime_secs
[1] 0

$seed
[1] 29

$build_tree_one_node
[1] FALSE

$mtries
[1] 5

$sample_rate
[1] 0.75

$binomial_double_trees
[1] FALSE

$col_sample_rate_change_per_level
[1] 1

$col_sample_rate_per_tree
[1] 1
```

$min_split_improvement
[1] 1e-05

$histogram_type
[1] "UniformAdaptive"

$categorical_encoding
[1] "Enum"

$calibrate_model
[1] FALSE

$distribution
[1] "multinomial"

$check_constant_response
[1] TRUE

$gainslift_bins
[1] -1

$x
```
 [1] "Albumin"              "Alkaline.phosphatase" "BUN"                  "Bilirubin..total"     "Calcium"              "Chloride"             "Creatinine"
 [8] "GOT..AST."            "GPT..ALT."            "Hb"                   "Hct"                  "PLT"                  "Phosphorus"
"Potassium"
[15] "Protein..total"       "Sodium"               "Uric.Acid"            "WBC"                  "hs.CRP.quantitation"
```

$y
[1] "death_inhosp"

XGBoost

$keep_cross_validation_models
[1] TRUE

$keep_cross_validation_predictions
[1] FALSE

$keep_cross_validation_fold_assignment
[1] FALSE

```
$score_each_iteration
[1] FALSE

$ignore_const_cols
[1] TRUE

$stopping_rounds
[1] 5

$stopping_metric
[1] "AUC"

$stopping_tolerance
[1] 1e-04

$max_runtime_secs
[1] 1.797693e+308

$seed
[1] 12345

$distribution
[1] "bernoulli"

$tweedie_power
[1] 1.5

$categorical_encoding
[1] "OneHotInternal"

$quiet_mode
[1] TRUE

$ntrees
[1] 10000

$max_depth
[1] 1

$min_rows
[1] 1

$min_child_weight
```

[1] 1

$learn_rate
[1] 0.05

$eta
[1] 0.05

$sample_rate
[1] 0.8

$subsample
[1] 0.8

$col_sample_rate
[1] 0.8

$colsample_bylevel
[1] 0.8

$col_sample_rate_per_tree
[1] 1

$colsample_bytree
[1] 1

$colsample_bynode
[1] 1

$max_abs_leafnode_pred
[1] 0

$max_delta_step
[1] 0

$score_tree_interval
[1] 10

$min_split_improvement
[1] 0

$gamma
[1] 0

```
$nthread
[1] -1

$build_tree_one_node
[1] FALSE

$calibrate_model
[1] FALSE

$max_bins
[1] 256

$max_leaves
[1] 0

$sample_type
[1] "uniform"

$normalize_type
[1] "tree"

$rate_drop
[1] 0

$one_drop
[1] FALSE

$skip_drop
[1] 0

$tree_method
[1] "exact"

$grow_policy
[1] "depthwise"

$booster
[1] "gbtree"

$reg_lambda
[1] 1
```

```
$reg_alpha
[1] 0

$dmatrix_type
[1] "dense"

$backend
[1] "cpu"

$gpu_id
[1] 0

$gainslift_bins
[1] -1

$x
 [1] "caseid"          "Albumin"         "Alkaline.phosphatase" "BUN"          "Bilirubin..total"   "Calcium"       "Chloride"
 [8] "Creatinine"      "GOT..AST."       "GPT..ALT."        "Hb"          "Hct"          "PLT"
"Phosphorus"
[15] "Potassium"       "Protein..total"   "Sodium"         "Uric.Acid"       "WBC"          "hs.CRP.quantitation"

$y
[1] "death_inhosp"
```

DNN

```
$keep_cross_validation_models
[1] TRUE

$keep_cross_validation_predictions
[1] FALSE

$keep_cross_validation_fold_assignment
[1] FALSE

$ignore_const_cols
[1] TRUE

$score_each_iteration
[1] FALSE

$balance_classes
[1] FALSE
```

```
$max_after_balance_size
[1] 5

$max_confusion_matrix_size
[1] 20

$overwrite_with_best_model
[1] TRUE

$use_all_factor_levels
[1] TRUE

$standardize
[1] TRUE

$activation
[1] "Tanh"

$hidden
[1] 30 30 30

$epochs
[1] 1

$train_samples_per_iteration
[1] -2

$target_ratio_comm_to_comp
[1] 0.05

$seed
[1] 1234747

$adaptive_rate
[1] TRUE

$rho
[1] 0.99

$epsilon
[1] 1e-08
```

```
$rate
[1] 0.005

$rate_annealing
[1] 1e-06

$rate_decay
[1] 1

$momentum_start
[1] 0

$momentum_ramp
[1] 1e+06

$momentum_stable
[1] 0

$nesterov_accelerated_gradient
[1] TRUE

$input_dropout_ratio
[1] 0

$l1
[1] 9.8e-05

$l2
[1] 2.1e-05

$max_w2
[1] 10

$initial_weight_distribution
[1] "UniformAdaptive"

$initial_weight_scale
[1] 1

$loss
[1] "Automatic"

$distribution
```

```
[1] "bernoulli"

$quantile_alpha
[1] 0.5

$tweedie_power
[1] 1.5

$huber_alpha
[1] 0.9

$score_interval
[1] 5

$score_training_samples
[1] 10000

$score_validation_samples
[1] 10000

$score_duty_cycle
[1] 0.025

$classification_stop
[1] 0

$regression_stop
[1] 1e-06

$stopping_rounds
[1] 2

$stopping_metric
[1] "logloss"

$stopping_tolerance
[1] 0.01

$max_runtime_secs
[1] 327.026

$score_validation_sampling
[1] "Uniform"
```

$diagnostics
[1] TRUE

$fast_mode
[1] TRUE

$force_load_balance
[1] TRUE

$variable_importances
[1] TRUE

$replicate_training_data
[1] TRUE

$single_node_mode
[1] FALSE

$shuffle_training_data
[1] FALSE

$missing_values_handling
[1] "MeanImputation"

$quiet_mode
[1] FALSE

$autoencoder
[1] FALSE

$sparse
[1] FALSE

$col_major
[1] FALSE

$average_activation
[1] 0

$sparsity_beta
[1] 0

$max_categorical_features
[1] 2147483647

$reproducible
[1] FALSE

$export_weights_and_biases
[1] FALSE

$mini_batch_size
[1] 1

$categorical_encoding
[1] "OneHotInternal"

$elastic_averaging
[1] FALSE

$elastic_averaging_moving_rate
[1] 0.9

$elastic_averaging_regularization
[1] 0.001

```
$x
  [1] "Albumin"              "Alkaline.phosphatase" "BUN"                  "Bilirubin..total"     "Calcium"              "Chloride"             "Creatinine"
  [8] "GOT..AST."            "GPT..ALT."            "Hb"                   "Hct"                  "PLT"                  "Phosphorus"
"Potassium"
[15] "Protein..total"       "Sodium"               "Uric.Acid"            "WBC"                  "hs.CRP.quantitation"
```

$y
[1] "death_inhosp"

GLM

$keep_cross_validation_models
[1] TRUE

$keep_cross_validation_predictions
[1] FALSE

$keep_cross_validation_fold_assignment

```
[1] FALSE

$fold_assignment
[1] "Random"

$ignore_const_cols
[1] TRUE

$score_each_iteration
[1] FALSE

$score_iteration_interval
[1] -1

$family
[1] "binomial"

$tweedie_variance_power
[1] 0

$tweedie_link_power
[1] 1

$theta
[1] 1e-10

$solver
[1] "IRLSM"

$lambda
[1] 0.000143091

$lambda_search
[1] FALSE

$early_stopping
[1] TRUE

$nlambdas
[1] -1

$standardize
[1] TRUE
```

```
$missing_values_handling
[1] "MeanImputation"

$compute_p_values
[1] FALSE

$remove_collinear_columns
[1] FALSE

$intercept
[1] TRUE

$non_negative
[1] FALSE

$max_iterations
[1] 50

$objective_epsilon
[1] 1e-04

$beta_epsilon
[1] 1e-04

$gradient_epsilon
[1] 1e-04

$link
[1] "logit"

$calc_like
[1] FALSE

$HGLM
[1] FALSE

$prior
[1] -1

$cold_start
[1] FALSE
```

```
$lambda_min_ratio
[1] 1e-04

$max_active_predictors
[1] 5000

$obj_reg
[1] 0.0003487967

$stopping_rounds
[1] 0

$stopping_metric
[1] "AUTO"

$stopping_tolerance
[1] 0.001

$balance_classes
[1] FALSE

$max_after_balance_size
[1] 5

$max_confusion_matrix_size
[1] 20

$max_runtime_secs
[1] 0

$x
 [1] "Albumin"              "Alkaline.phosphatase" "BUN"                  "Bilirubin..total"     "Calcium"              "Chloride"             "Creatinine"
 [8] "GOT..AST."            "GPT..ALT."            "Hb"                   "Hct"                  "PLT"                  "Phosphorus"
"Potassium"
[15] "Protein..total"       "Sodium"               "Uric.Acid"            "WBC"                  "hs.CRP.quantitation"

$y
[1] "death_inhosp"
```

Supplementary Material S3: A calibration plot comparing the predicted probability computed by the random forest model with the fraction of observed outcome.

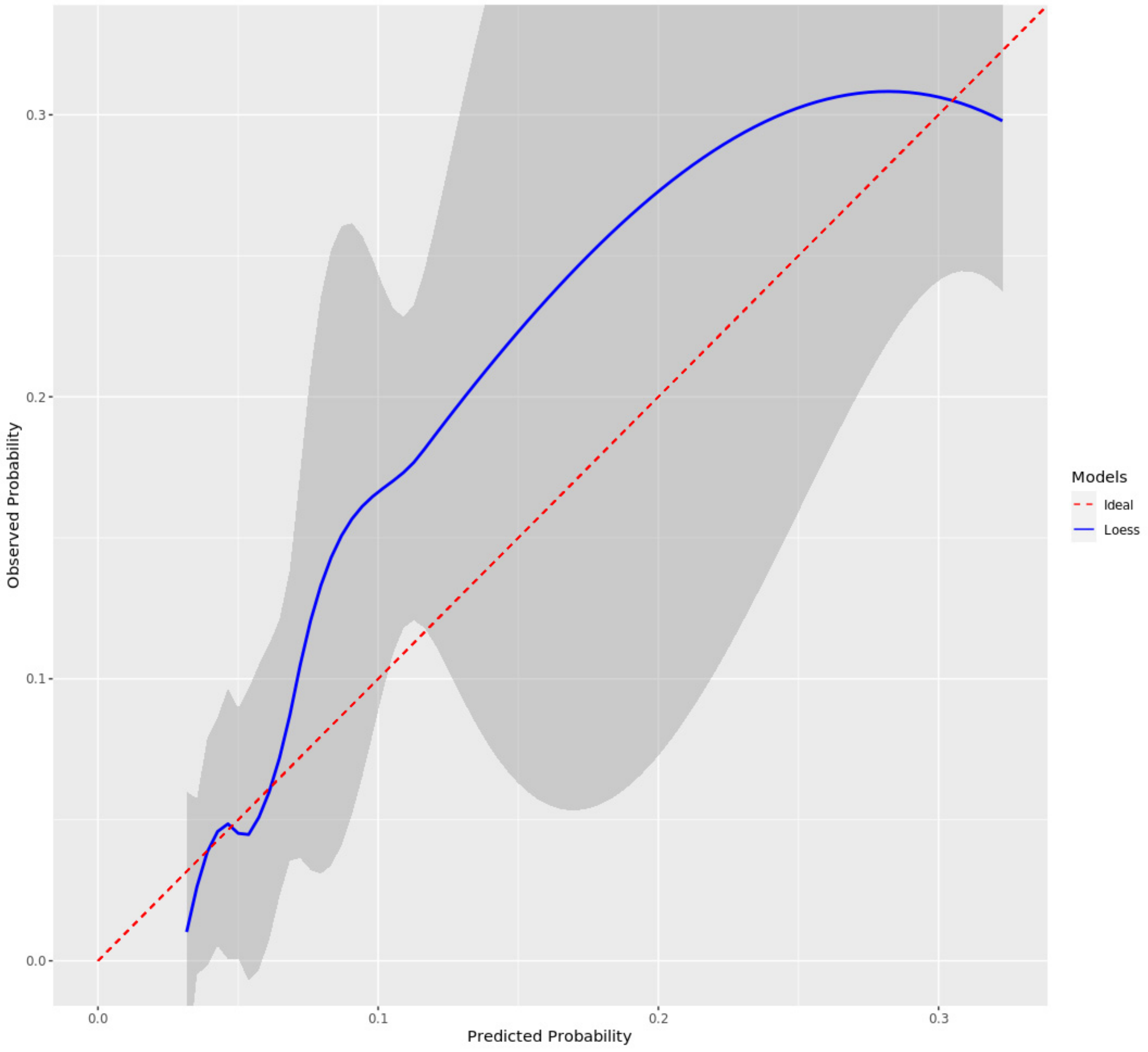

Linear Model:

Intercept	Gradient
0.001901101	0.981541599

Hosmer–Lemeshow test:

	Xsquared	df	pvalue
1	10.51912	8	0.2304626
